# The Isolate *Pseudomonas multiresinivorans* QL-9a Quenches the Quorum Sensing Signal and Suppresses Plant Soft Rot Disease

**DOI:** 10.3390/plants12173037

**Published:** 2023-08-24

**Authors:** Siqi Liu, Xixian Zhu, Zhenchen Yan, Hui Liu, Lianhui Zhang, Wenjuan Chen, Shaohua Chen

**Affiliations:** 1 National Key Laboratory of Green Pesticide, Guangdong Province Key Laboratory of Microbial Signals and Disease Control, Integrative Microbiology Research Centre, South China Agricultural University, Guangzhou 510642, China; 2 Guangdong Laboratory for Lingnan Modern Agriculture, College of Plant Protection, South China Agricultural University, Guangzhou 510642, China

**Keywords:** biocontrol, *N*-(-3-oxohexanoyl)-*L*-homoserine lactone, *Pseudomonas multiresinivorans* QL-9a, quorum quenching

## Abstract

Quorum sensing (QS) is a communication mechanism used among microorganisms that regulate the population density and behavior by sensing the concentration of signaling molecules. Quorum quenching (QQ), a novel, eco-friendly, and efficient method for disease control, interferes with QS by disturbing the production and enzymatic degradation of signaling molecules, blocking communication among microorganisms, and thus has deep potential for use in plant disease control. *Pectobacterium carotovorum* can cause bacterial soft rot, resulting in yield reduction in a variety of crops worldwide, and can be mediated and regulated by the *N*-acyl homoserine lactones (AHLs), which are typical signaling molecules. In this study, a novel quenching strain of *Pseudomonas multiresinivorans* QL-9a was isolated and characterized, and it showed excellent degradation ability against AHLs, degrading 98.20% of *N*-(-3-oxohexanoyl)-*L*-homoserine lactone (OHHL) within 48 h. Based on the results of the gas chromatography–mass spectrometer (GC–MS) analysis, a possible pathway was proposed to decompose OHHL into fatty acids and homoserine lactone, in which AHL acylase was involved. Additionally, it has been demonstrated that the QL-9a strain and its crude enzyme are promising biocontrol agents that can considerably reduce the severity of the soft rot disease brought on by *P. carotovorum*, consequently preventing the maceration of a variety of host plant tissues. All of these results suggest promising applications of the QL-9a strain and its crude enzyme in the control of various plant diseases mediated by AHLs.

## 1. Introduction

Quorum sensing (QS) is a microbial cell-to-cell communication mechanism by which bacteria detect and recognize the levels of various signaling molecules in the environment to regulate population density and behavior [1,2,3]. When the signaling molecules reach the threshold, specific receptor proteins can be bound and activate related genes for expression [4]. This regulates various physiological activities, such as the luminescence activity of *Vibrio felis*, the formation of biofilm, the expression of virulence factors, and the production of antibiotics by bacteria [5,6].

There are various types of signaling molecules, such as the self-inducible factor, oligopeptides [7], *N*-acyl homoserine lactones (AHLs) [8], 2-heptyl-3-hydroxy-4-quinolone [9], and *cis*-11-methyl-2-dodecenoic acid [10]. AHLs, also referred to as autoinducer-1 (AI-1) [11], are the most prevalent signaling molecules in the QS system [12,13]. They are synthesized by LuxI or its homologs in various bacteria [14]. More than 30 kinds of AHL family have been identified from more than 70 bacteria, such as *Pseudomonas* spp., *Burkholderia* spp. [15], *Pantoea* spp. [16], *Erwinia* spp. [17], and many other plant pathogens process the AHL-regulated QS system, *Pectobacterium carotovorum* being one of them [18]. It is a significant inducer of bacterial soft rot disease, which is among the top ten bacterial diseases, resulting in yield reductions of crops (e.g., carrot, cabbage, and potato), and is one of the most common diseases in potatoes [19,20]. *P. carotovorum* synthesizes and secretes a series of cell-wall-degrading enzymes (PCWDE) [21], including cellulase and pectin lyase, to promote the destruction of plant tissues. It impregnates the host plant, eventually leading to soft rot and collapse. Research shows that the production of PCWDE is strictly regulated by *N*-(3-oxohexanoyl)-*L*-homoserine lactone (OHHL) [22,23].

The main methods used to control bacterial diseases caused by *P. carotovorum* are the use of chemical pesticides and antibiotics, such as copper sulfate, dimethyl ammonium chloride, antibiotics (streptomycin and its derivatives), and other chemical control strategies [24]. However, the massive use of chemical pesticides and antibiotics has led to the development of bacterial resistance, while ecological diversity, food safety, and other issues are also becoming increasingly apparent, so the development of a new green control strategy is urgently needed [25,26,27].

Quorum quenching (QQ) is emerging as a high-potential disease management strategy in contemporary agriculture. QQ is designed to disturb QS in different ways, such as inhibiting the production of signaling molecules, promoting their degradation/modification, and competitively inhibiting binding between signals and receptors [28,29]. Thereinto, QS can be inhibited at the stages of signal production, transmission, and perception. Signal production suppression can be achieved by the expression product of the signal synthetase [30,31]. During the diffusion of signal molecules, specific substances, such as quenching enzymes, can degrade the QS signaling molecule and lead to attenuation of QS-regulated traits [32]. Signaling molecule analogs compete with signaling molecules to prevent the binding of the receptor protein [33,34]. Quenching enzymes, i.e., group quenching technology, can achieve this purpose without killing the microorganisms, consequently preventing the emergence of pathogenic bacteria resistance, which has developed as a green and efficient control strategy with broad application prospects [35].

In the present study, we isolated and characterized a quorum quencher of AHLs that was identified as *Pseudomonas multiresinivorans* QL-9a. Through a study of the degradation ability, products, and degradation mechanism of QL-9a, it was shown that the QL-9a strain efficiently degrades AHLs. The biological control effect of QL-9a and its crude enzyme on bacterial soft rot caused by *P. carotovorum* was also investigated. This study offers an interesting perspective on the pathogenic regulatory mechanism of pathogens mediated by AHLs. Interestingly, studies have shown that *P. multiresinivorans* has the characteristic of resistance to petroleum hydrocarbons (such as n-heptane, n-decane, n-pentadecane, n-hexadecane, o-xylene, etc.) and can degrade diesel [36], but *P. multiresinivorans* has rarely been reported in the prevention and control of plant diseases.

## 2. Results

### 2.1. Identification and Characterization of the QL-9a Strain

After the isolation and screening of AHL degradation strains, the reporter strain NT1 was used as a biosensor (Figure S1), and the preliminary degradation effect was determined. Fourteen morphologically different strains capable of degrading AHL were attained by an enrichment culture. The *P. multiresinivorans* QL-9a strain with high AHL degradation activity was selected and used in further studies.

**Figure 1:**
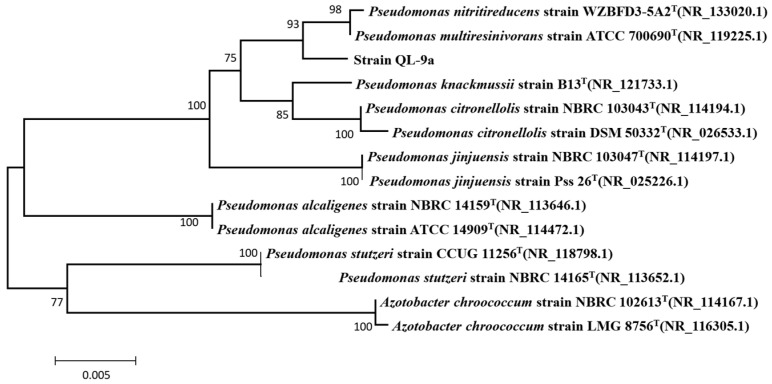
Phylogenetic tree based on 16S rDNA sequences of *Pseudomonas multiresinivorans* strain QL-9a. Numbers in parentheses represent GenBank accession numbers. Numbers at nodes indicate bootstrap values. The bar represents sequence divergence.

The colonies of QL-9a were flat and light green in color, with smooth opaque surfaces and blurred edges (Figure S2a). The photo taken by optical microscopy is displayed in Figure S2b. The strain was rod-shaped and had a flagellum, according to the scanning electron microscopy results (Figure S2c). It tested positive for oxidase and nitrate reductase and utilized D-glucose and citrate; it tested negative in indole, methyl red, hydrogen sulfide, D-fructose fermentation, and V-P assay (Table S1). The phylogenetic relationship of QL-9a and other strains is displayed in the phylogenetic tree generated by MEGA 11.0 (Figure 1). The comprehensive results show that the QL-9a strain belongs to *P. multiresinivorans*. It was stored in Guangdong Microbial Culture Collection Center under the registration number GDMCC No: 60761.

### 2.2. Growth and Degradability Test

**Figure 2:**
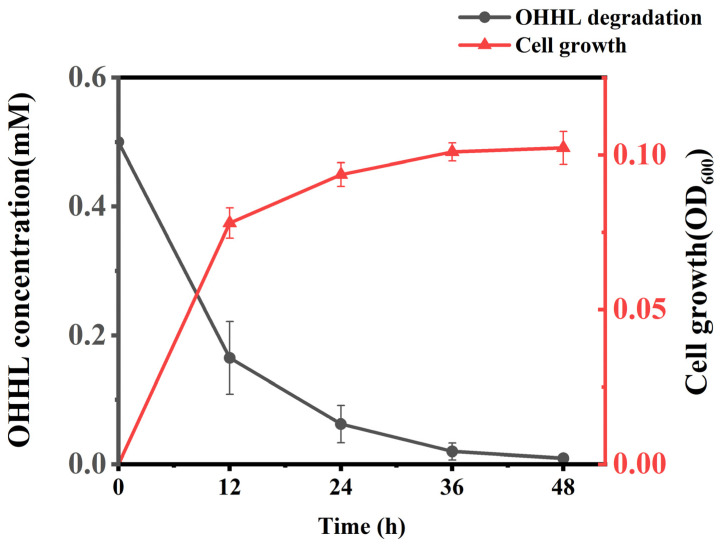
Degradation of *N*-(3-oxohexanoyl)-*L*-homoserine lactone (OHHL) during the growth of *Pseudomonas multiresinivorans* strain QL-9a. Each experiment was conducted with three replicates. Bars indicate the standard deviation of the mean.

The growth of QL-9a and the degradation of OHHL (0.5 mmol·L^−1^) were studied in MSM medium. As is shown in Figure 2, 98.20% of OHHL was degraded at 48 h post-inoculation. The HPLC results are shown in Figure S3, from which it can be seen that the amount of AHL decreased with time. The strain’s growth was positively correlated to the OHHL’s decrease. In the presence of OHHL, the growth curve expanded without a lagging phase and quickly transitioned into the logarithmic phase of development. Moreover, the strain’s logarithmic growth phase was within 12 h, during which time it degraded OHHL at the fastest rate.

### 2.3. Biological Control Effect against Z3-3

**Figure 3:**
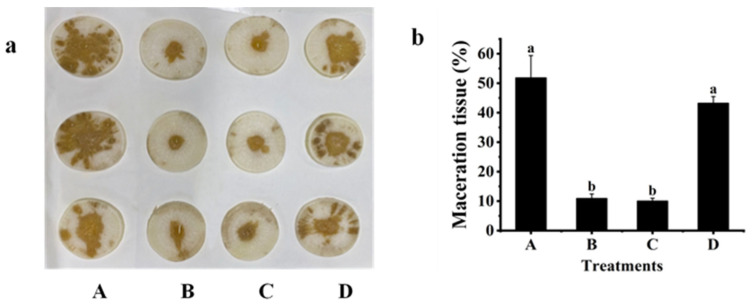
Maceration attenuating test of *Pseudomonas multiresinivorans* QL-9a against soft rot disease pathogen *Pectobacterium carotovorum* Z3-3 on White radish (*Raphanus sativus*). *Bacillus thuringiensis* subsp. *israelensis* B23 and *Escherichia coli* PLG107 served as positive and negative controls, respectively. (**a**) Panel A, Z3-3 alone on plant slices; Panel B, Z3-3 + QL-9a; Panel C, Z3-3 + B23; Panel D, Z3-3 + PLG107. (**b**) Maceration tissue in each treatment. Experimental data were analyzed by one-way analysis of variance (ANOVA), and means were compared by Bonferroni’s multiple comparison test in GraphPad Prism (Version 6.0). Experiments were arranged as a completely randomized design, and *p*-values < 0.05 were considered statistically significant. Different letters indicate significant differences (*p* < 0.05).

**Figure 4:**
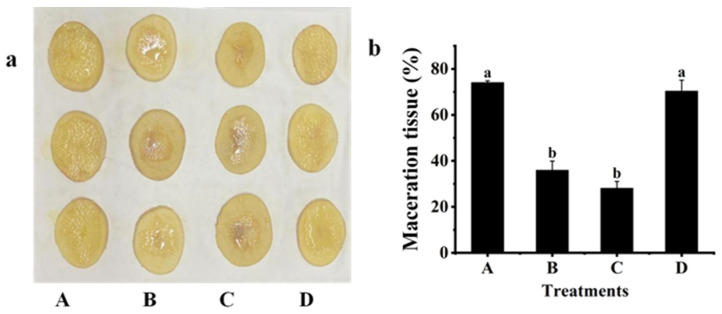
Maceration attenuating test of *Pseudomonas multiresinivorans* QL-9a against soft rot disease pathogen *Pectobacterium carotovorum* Z3-3 on potato (*Solanum tuberosum* L.). *Bacillus thuringiensis* subsp. *israelensis* B23 and *Escherichia coli* PLG107 served as positive and negative controls, respectively. (**a**) Panel A, Z3-3 alone on plant slices; Panel B, Z3-3 + QL-9a; Panel C, Z3-3 + B23; Panel D, Z3-3 + PLG107. (**b**) Maceration tissue in each treatment. Experimental data were analyzed by one-way analysis of variance (ANOVA), and means were compared by Bonferroni’s multiple comparison test in GraphPad Prism (Version 6.0). Experiments were arranged as a completely randomized design, and *p*-values < 0.05 were considered statistically significant. Different letters indicate significant differences (*p* < 0.05).

**Figure 5:**
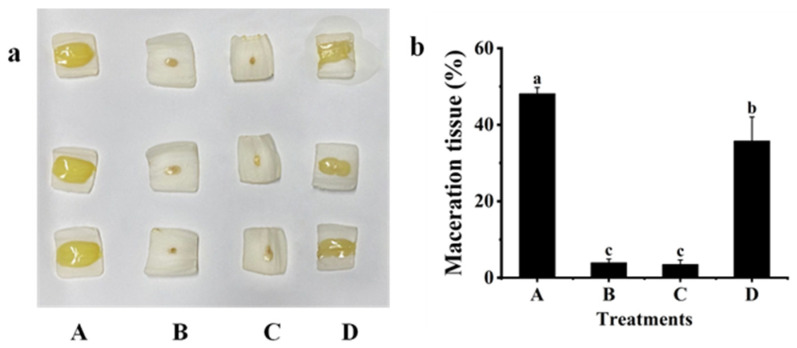
Maceration attenuating test of *Pseudomonas multiresinivorans* QL-9a against soft rot disease pathogen *Pectobacterium carotovorum* Z3-3 on Chinese cabbage (*Brassica pekinensis* (Lour.) Rupr.). *Bacillus thuringiensis* subsp. *israelensis* B23 and *Escherichia coli* PLG107 served as positive and negative controls, respectively. (**a**) Panel A, Z3-3 alone on plant slices; Panel B, Z3-3 + QL-9a; Panel C, Z3-3 + B23; Panel D, Z3-3 + PLG107. (**b**) Maceration tissue in each treatment. Experimental data were analyzed by one-way analysis of variance (ANOVA), and means were compared by Bonferroni’s multiple comparison test in GraphPad Prism (Version 6.0). Experiments were arranged as a completely randomized design, and *p*-values < 0.05 were considered statistically significant. Different letters indicate significant differences (*p* < 0.05).

The biological control effect of Z3-3 by strain QL-9a was evaluated on white radish (*R. sativus*) (Figure 3), potato (*S. tuberosum* L.) (Figure 4), and Chinese cabbage (*B. pekinensis* (Lour.) Rupr (Figure 5), and potato stem (Figure S4). The results demonstrated that soft rot disease developed severely on plant slices inoculated with single Z3-3 and on those co-inoculated with Z3-3 and *E. coli* PLG107. Co-inoculation with QL-9a and Z3-3 gave similar results to co-inoculation with the B23 strain and Z3-3, and both groups showed significant attenuation of soft rot symptoms, which was indicated by the area of maceration. These findings imply that the QL-9a strain is a potent quencher of the Z3-3 pathogen and may be capable of suppressing other AHL-dependent bacterial pathogens.

### 2.4. Degradation Products and the Pathway of OHHL

**Figure 6:**
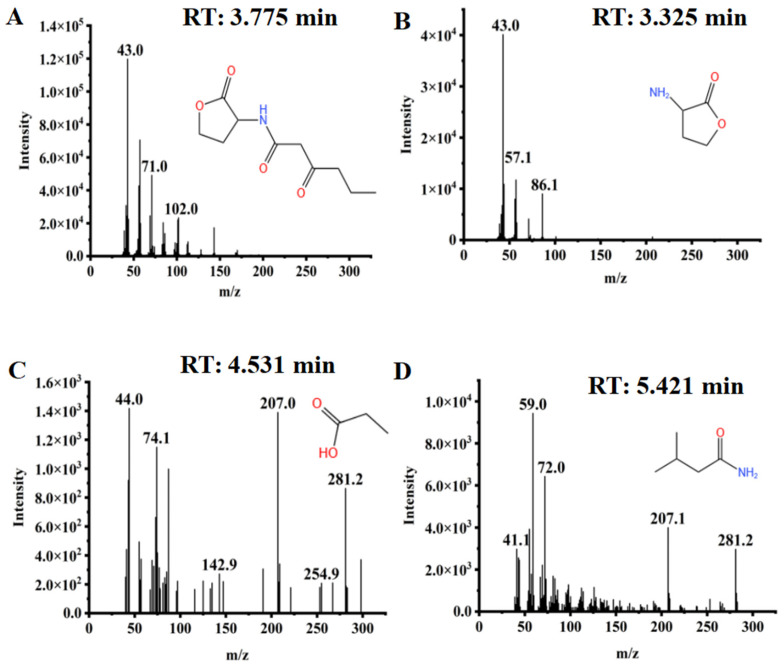
The mass spectra of the degradation products with the authentic standard compounds of the National Institute of Standards and Technology (NIST, USA) library database. (**A**) *N*-(3-oxohexanoyl)-*L*-homoserine lactone; (**B**) Homoserine lactone, (**C**) Propanoic acid; (**D**) 3-Methyl-butanamide.

**Figure 7:**
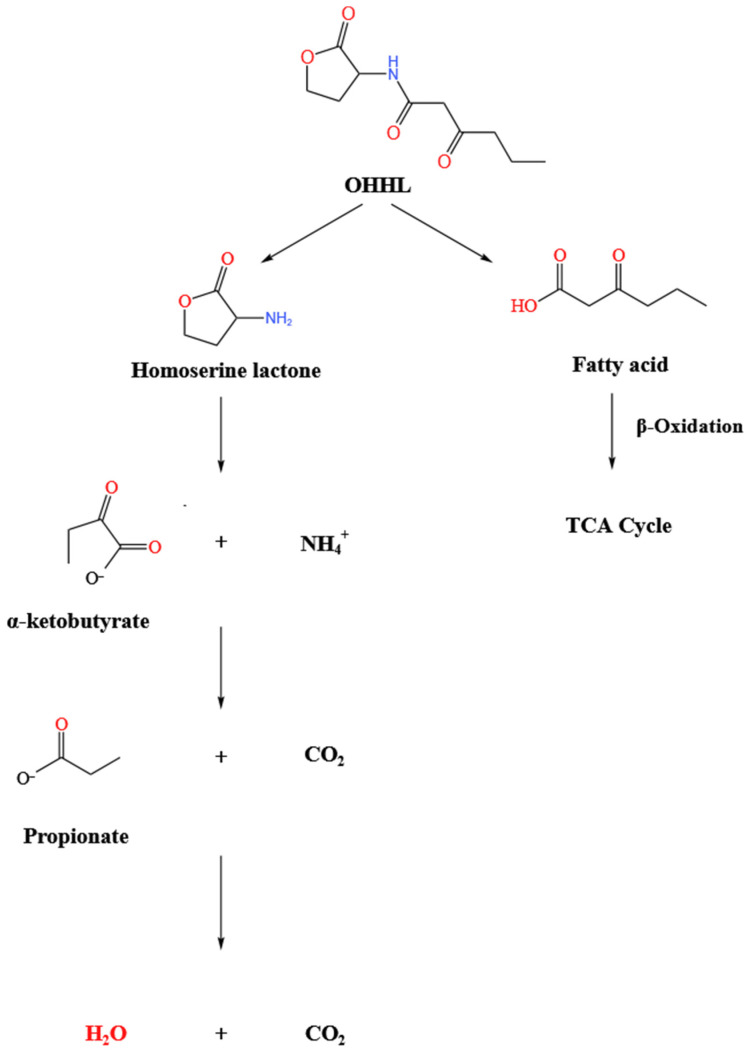
Proposed *N*-(3-oxohexanoyl)-*L*-homoserine lactone (OHHL) degradation pathway in *Pseudomonas multiresinivorans* QL-9a. OHHL could be degraded first by cleavage of its amide bond, followed by hydrolysis of the lactonic ring and subsequent metabolism.

To elucidate the pathway by which QL-9a degrades OHHL, samples were taken at the same time interval in MSM medium supplemented with OHHL (0.5 mmol·L^−1^). Figure 6 depicts the GC-MS findings. With a clear peak at a retention time (RT) of 3.775 min and a distinctive mass fragment at *m*/*z* 43.0, compound A was identified as OHHL. A significant amount of compound B was detected at an RT of 3.325 min, showing characteristic ionic fragmentation at *m*/*z* = 43.0 and a major fragmentation ion at *m*/*z* = 57.1 and was eventually characterized as a homoserine lactone. Compound C was detected at an RT of 4.531 min, eluting down to show a prominent fragment ion at *m*/*z* = 44.0, and was identified as propionic acid. Other metabolites of OHHL were also detected, and all were transiently present (Figure S5). Based on the chemical structure of OHHL and the intermediate metabolites, the metabolic pathway of OHHL was proposed, indicating that the amide bond of OHHL is cleaved to produce homoserine lactone and fatty acids, and homoserine lactone is further metabolized, while fatty acids are oxidized and used as energy substances for bacterial physiological activities (Figure 7).

### 2.5. Antagonism Test of the QL-9a Strain against Z3-3

To investigate whether the QL-9a strain presents an antagonistic effect to the pathogenic Z3-3 bacteria, bacterial suspensions of the QL-9a strain were injected into wells of solid, flat LB plates containing pathogenic Z3-3 bacteria. The results showed that no inhibition zone was produced when the QL-9a strain and pathogenic Z3-3 bacteria were co-cultured. Thus, the QL-9a strain did not show an antagonistic effect against the Z3-3 pathogen (Figure S6).

### 2.6. Biocontrol Effect of Crude Enzymes

**Figure 8:**
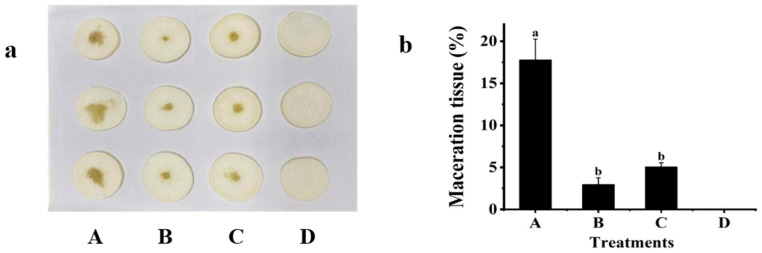
Preliminary biocontrol test of crude enzymes of *Pseudomonas multiresinivorans* QL-9a against *Pectobacterium carotovorum* Z3-3. (**a**) Panel A: Z3-3 alone on plant slices; Panel B: Z3-3 + Intracellular enzyme; Panel C: Z3-3 + Extracellular enzyme; Panel D: Sterile water. (**b**) Maceration tissue in each treatment. Experimental data were analyzed by one-way analysis of variance (ANOVA), and means were compared by Bonferroni’s multiple comparison test in GraphPad Prism (Version 6.0). Experiments were arranged as a completely randomized design, and *p*-values < 0.05 were considered statistically significant. Different letters indicate significant differences (*p* < 0.05).

Plant tissue samples were evaluated in vitro to confirm the ability of the QL-9a strain’s crude enzyme to biocontrol Z3-3. The pathogenic Z3-3 bacteria (alone), intracellular enzyme, and extracellular enzyme were inoculated in the tubers of white radish mixed with Z3-3 and incubated for 24 h. Figure 8 shows that the maceration area was significantly smaller in the crude-enzyme-treated group compared with the Z3-3-treated group. These findings demonstrated that the virulence of Z3-3 to white radish effectively attenuates tissue maceration by the AHL-hydrolyzing enzymes contained in the QL-9a suspension.

### 2.7. Acylase Activity Assay

The degradation intermediate identified by GC-MS indicates QL-9a produces AHL acylase. In this section, the acylase activity of intra/extracellular enzymes of QL-9a was tested. The outcome of the color reaction is displayed in Figure S7. When compared to the control group, solutions of both the intracellular and extracellular enzyme groups formed the reductive substance 2-nitro-5-thiobenzoic acid (TNB), which showed up as a yellow color with an absorbance peak at 412 nm. This indicates that the QL-9a strain has acylase activity and can degrade OHHL to fatty acids and homoserine lactones.

## 3. Materials and Methods

### 3.1. Plants and Chemicals

The AHL signaling molecule *N*-(3-oxohexanoyl)-*L*-homoserine lactone (OHHL) was purchased from Shanghai UDChem Technology Co., Ltd. (Shanghai, China). Chinese cabbage (*Brassica pekinensis* (Lour.) Rupr.), white radish (*Raphanus sativus*), and potato (*Solanum tuberosum* L.) were purchased from the Guangzhou local market (Guangzhou, China).

### 3.2. Strains and Media

*Agrobacterium tumefaciens* NT1, *Escherichia coli* PLG107, *Bacillus thuringiensis* subsp. *israelensis* B23, *Pseudomonas multiresinivorans* QL-9a, and *P. carotovorum* Z3-3 (soft rot disease pathogen) were preserved at the Integrated Microbial Research Center, South China Agricultural University, Guangdong Province, China. Luria–Bertani (LB) medium contains yeast extract, tryptone, and NaCl at 5.0, 10.0, and 10 g/L, respectively). Mineral salt medium (MSM) contains the following ingredients in 1000 mL distilled water: KH_2_PO_4_, 1.5 g; (NH_4_)_2_ SO_4_·12H_2_O, 2.0 g; FeSO_4_, 0.001 g; CaCl_2_·2H_2_O, 0.01 g; Na_2_HPO_4_, 1.5 g; and MgSO_4_·7H_2_O, 0.2 g.

### 3.3. Isolation of QL-9a

The bacteria were isolated in soil that was taken from the Guangzhou trial field, Guangdong Province, China. In order to isolate bacteria that break down AHLs, OHHL at a final concentration of 50 μM was added to the MSM medium along with 5 g of soil sample in the medium and incubated for 7 days (30 °C, 200 rpm) and then transferred to a second batch of MSM medium with 100 μM OHHL at 10% inoculum. After 7 days of incubation under the same conditions, a 10% inoculum was transferred to MSM medium at 200 μM OHHL and incubated for another 7 days. The concentration of OHHL in the medium was then increased. Afterward, the isolation and purification of the strains were carried out by dilution and plate streaking [37]. AHL’s biosensor, *A. tumefaciens* NT1 [38], was used to screen strains with the highest degradation activities. In this process, β-galactosidase was generated and could cleave X-gal (5-bromo-4-chloro-3-indolyl-β-D-galactoside) to form a blue substrate. The content of AHLs in the sample was determined based on the distance from the top of the agar strip to the blue color [8].

### 3.4. Identification of the QL-9a Strain

The isolated QL-9a strain was incubated for 24 h at 30 °C on solid LB plates. Optical microscopy (BH-2 Olympus, Tokyo, Japan) was used to observe cell morphology, and scanning electron microscopy (XL-30 ESEM, Philips Optoelectronics Co., Ltd., Amsterdam, The Netherlands) was used to observe the morphology of cells on slices. The target strain’s 16S rDNA was amplified using universal primers (27F: 5-AGAGTTTGAT CCTGGCTCAG-3; 1492R: 5-GGTTACCTT GTTACGACTT-3), and the amplified products were sequenced. 16S rDNA sequences from type strains were downloaded from the NCBI database (http://www.NCBI.nlm.nih.gov/, accessed on 15 June 2023). MEGA 11.0 software was then used to generate a phylogenetic tree after aligning the sequences by ClustalW [39,40].

### 3.5. Determination of OHHL Degradation Activity by HPLC

The preserved QL-9a strain was inoculated on solid LB culture plates at 30 °C for 24 h. Individual colonies on the plates were picked and then incubated in liquid LB medium at 30 °C and 200 rpm until the OD_600_ reached 1. Subsequently, the seed culture of QL-9a (1 mL) was added to MSM medium (50 mL) containing OHHL at a concentration of 0.5 mmol·L^−1^ and incubated at 30 °C and 200 rpm. Samples were collected at 0 h, 12 h, 24 h, 36 h, and 48 h after inoculation. The OD_600_ values were measured using a UV–Vis spectrophotometer, and the residual amount of OHHL was determined by high-performance liquid chromatography (HPLC) (Waters, Milford, MA, USA). The samples prepared for HPLC were extracted from MSM media by ethyl acetate, dried up, and dissolved in 2 mL chromatographic grade methanol (BCR, Ann Arbor, MI, USA), and then the solution was passed through a 0.22 μm organic filter membrane into a vial. The mobile phase of HPLC contained 30% acetonitrile and 70% water (*ν*:*ν*), the injection volume was 20 μL, the chromatographic column was a Kinetex EVO C_18_ Reverse Phase Chromatography Column, and the detection wavelength was 210 nm [8].

### 3.6. Effect of the QL-9a Strain on Soft Rot Disease Caused by Z3-3

The following in vitro assay was carried out to investigate the ability of QL-9a to control Z3-3 causing soft rot diseases [28]. *P. carotovorum* Z3-3 was inoculated as a soft rot pathogen, and *P. multiresinivorans* QL-9 was used as a quorum quenching strain. In addition, *B. thuringiensis* subsp. *israelensis* B23, a well-known strain that degrades AHLs, served as a positive control, whereas *E. coli* PLG107 served as a negative control. White radish (*R. sativus*), potato (*S. tuberosum* L.), and Chinese cabbage (*B. pekinensis* (Lour.) Rupr) were used as experimental materials. After the host plants were cleaned and surface sterilized in 75% ethanol, they were sliced into portions with a rough thickness of 0.4 cm. The OD_600_ of the liquid culture of PLG107, B23, QL-9a, and Z3-3 was diluted to 1 by adding sterile water, followed by inoculation on experimental plants. The inoculum for the four groups is Z3-3/LB media, Z3-3/QL-9a mixed culture, Z3-3/B23 mixed culture, and Z3-3/PLG107 mixed culture, respectively, all at a ratio of 1:9 and inoculation amount of 5 μL. Each treatment was conducted in triplicate. After inoculation, the host plants were incubated at 28 °C for 24 h. The maceration area and slice area were determined by ImageJ. The percentage of maceration was calculated by the ratio of the maceration area to the slice area.

### 3.7. Identification of OHHL Degradation Products by GC-MS

QL-9a was inoculated in MSM medium containing OHHL at a concentration of 0.5 mmol·L^−1^, incubated at 30 °C and 200 rpm, and sampled at 12 h intervals, with three samples collected simultaneously from each group. The sample processing method is mentioned in Section 3.5. and was subjected to gas chromatography–mass spectrometry (GC–MS) (Agilent 6890 N/5975, Santa Clara, CA, USA) for degradation product detection. The following were the detection requirements: an Agilent HP-5MS quartz capillary column (30 m × 250 μm × 0.25 μm) with high-purity helium as the carrier gas, flowing at a rate of 1.0 mL/min. The temperature increased from 200 °C to 280 °C at a rate of 25 °C per minute. Electron energy of 70 eV, a split injection volume of 1 μL, and a split ratio of 5:1 was used. The quadrupole was heated to 150 °C, while the ion source was heated to 230 °C [21].

### 3.8. Antagonism Test of the QL-9a Strain

The following method was used to investigate whether the QL-9a strain interacts antagonistically with pathogenic bacteria. Melted LB and Z3-3 suspension were mixed together until the plates were solidified. The LB agar plates containing the pathogen were perforated with a sterilized perforator, and 20 μL QL-9a liquid culture was injected into the wells, while 20 μL sterile water was injected into the wells of the control group. Each experiment was conducted in triplicate. After 24 h of incubation at 30 °C, we observed the development of transparent circles to evaluate the antagonistic effect.

### 3.9. Effect of the QL-9a Crude Enzyme on Z3-3

The crude enzyme of the QL-9a strain was prepared according to the method of Ye et al. [41]. The QL-9a strain was cultured in LB media overnight and centrifuged (10,000 rpm, 4 °C, 10 min), and the supernatant was used as an extracellular enzyme. Cells were washed three times, resuspended in PBS, and disrupted by sonication at 4 °C. The homogenate was then centrifuged at 10,000 rpm for 10 min at 4 °C. The harvested supernatant was used as a source of intracellular enzymes. Each set of experiments was repeated three times. The experiments consisted of four groups: (1) Z3-3-treated radish slices; (2) radish slices treated with the QL-9a extracellular enzyme and Z3-3; (3) radish slices treated with the QL-9a intracellular enzyme and Z3-3; and (4) radish slices treated with sterile water as a blank control. Each treatment had three replicates, and the severity of the plant slice incidence was measured.

### 3.10. Acylase Activity Assay

The acylase activity of QL-9a was analyzed using an acylase activity assay kit (Solarbio, Beijing Solarbio Science & Technology Co., Ltd., Beijing, China). The QL-9a strain was incubated overnight in liquid LB media until OD_600_ reached 1, and the intracellular and extracellular enzymes were collected separately, as described in Experiment 3.9. Acylase catalyzes the reduction of 5,5′-dithiobis-(2-nitro-benzoic acid) (DTNB) to form 2-nitro-5-thiobenzoic acid (TNB), the solution of which turns yellow with an absorption peak at 412 nm. OD_412_ of each treatment was quantified using a UV–Vis spectrophotometer [42].

### 3.11. Data Analysis

All experiments were completely randomized. Data are presented as the mean ± standard deviation. Experimental data were analyzed by one-way analysis of variance (ANOVA), and data from the biocontrol control effect test were compared for means by Bonferroni multiple comparison test in GraphPad Prism (version 6.0). Data for growth and degradability tests were compared for means by Bonferroni multiple comparison test in SPSS25. Graphs were plotted using ORIGIN 2019. The threshold for statistical significance was set at *p* < 0.05.

## 4. Discussion

Due to their high efficiency in disease control, pesticides, and antibiotics have been widely used in agricultural production, and serious environmental problems have also emerged. Moreover, they are toxic to non-target organisms, and the use of large amounts of pesticides creates problems such as environmental pollution and food safety [43,44]. In addition, the therapeutic effect of antibiotics on drug-resistant pathogens is becoming weaker and weaker, while the emergence of drug-resistant bacteria poses a threat to human health [45]. Thus, it is crucial to create innovative, safe, and effective disease control strategies.

QS is associated with the expression of virulence factors and pathogenic genes, biofilm formation, the production of extracellular polysaccharides, and other biological functions that affect the pathogenic ability of pathogenic bacteria [46]. QQ is a disease control strategy based on QS, viz., the conversion of pathogenic to nonpathogenic bacteria by quenching the signaling molecules of pathogenic bacteria for the purpose of controlling the concentration of signaling molecules below the threshold of activation of pathogenic genes. Several studies have shown that strains with AHLs quenching activity include *Bacillus* sp. [47], *Streptomyces* sp., *Ralstonia* sp. [48], *Rhodococcus* sp. [49], *Pseudomonas* sp. [50], *Rhizobium* sp., *Ochrobactrum* sp., and *Azospirillum* sp. [51]. Previous studies have shown that only three types of quenching enzymes are produced by induction quenching of different populations: lactase [52], acylase [53], and oxidoreductase [54].

In this study, QL-9a, capable of efficiently degrading AHL signaling molecules, was shown to be *P. multiresinivorans*. Recent studies have shown that a variety of *Pseudomonas* sp. are widely used as biological control agents and have the advantage of colonizing plant roots and inhibiting a variety of plant diseases [55,56,57,58,59]. They prevent and control plant diseases by producing pathogen-resisting agents, such as antibacterial metabolites, proteases, lipases, and chitinases [60,61,62]. The *P. multiresinivorans* QL-9a strain tested in this study enlarges the reservoir of bacteria used as biological control agents. Future research can be deepened into the molecular mechanism to better understand the role of *Pseudomonas* sp. in the field of biocontrol.

Strain QL-9a has the ability to efficiently quench the quorum-sensing signal molecules AHLs. In the acylase test, QL-9 was shown to have acylase activity. Unlike lactonase, acylase does not repair the structure of the lactone ring in acidic conditions. As no persistent products were observed, it is hypothesized that strain QL-9a possesses an intact mechanism for OHHL degradation. In addition to the degradation ability of QL-9a on AHL signaling molecules, the biocontrol effect of QL-9a on Z3-3 was investigated, and the results showed that the maceration area and pathogenesis index of plant sections using QL-9a were significantly reduced. That is, QL-9a can control bacterial soft rot regulated by AHL.

These studies reveal the biochemical basis of efficient AHL degradation by QL-9a and provide a new option for the development of new disease control strategies. However, future studies on the genetic characteristics and genome-wide analysis of AHL degradation by the QL-9a strain still need to be further explored so that it can be used in actual production practices.

## 5. Conclusions

In conclusion, we discovered a new quorum quencher, *P. multiresinivorans* QL-9a, which, along with its enzyme, has the potential to act as a biocontrol agent and reduce the severity of Z3-3-induced soft rot. The QQ strain QL-9a has an excellent AHL degradation capacity and harbors the metabolic pathway for the complete degradation and metabolism of OHHL. Based on the results of the acylase test, we further confirmed the acylase activity of strain QL-9a and proposed a pathway for the biodegradation of AHL by QL-9a. Our findings uncover the biochemical characteristics of a highly efficient AHL-degrading bacterial isolate and provide a useful strain/enzyme with enormous potential for the biocontrol of soft rot diseases caused by AHL-dependent bacterial pathogens.

## Data Availability

Not applicable.

## Supplementary Materials

The following supporting information can be downloaded at: https://www.mdpi.com/article/10.3390/plants12173037/s1, Figure S1. Degradation of *N*-(3-oxohexanoyl)-*L*-homoserine lactone (OHHL) by strain QL-9a. A: Negative control: only containing OHHL (10 μmol·L^−1^). B: Degradation of OHHL (10 μmol·L^−1^) by strain QL-9a. The diffusion length of OHHL and the blue colonies increase with the increase of OHHL concentration. Each colony corresponds to the biosensor strain *Agrobacterium tumefaciens* NT1. Figure S2. Morphological characteristics of *Pseudomonas multiresinivorans* strain QL-9a. (a) Colonial morphology of strain QL-9a; (b) Cell morphology observed under an optical microscope; (c) Cell morphology observed under the scanning electron microscope (3000×). Figure S3. The remaining amount of *N*-(3-oxohexanoyl)-*L*-homoserine lactone (OHHL) at different time intervals was determined by high-performance liquid chromatography (HPLC): (A) Mineral salt medium (MSM) with OHHL alone as a control. OHHL degradation by the QL-9a strain at 12 h (B), 24 h (C), 36 h (D), and 48 h (E), respectively. Figure S4. Maceration attenuating test of *Pseudomonas multiresinivorans* QL-9a against soft rot disease pathogen *Pectobacterium carotovorum* Z3-3 on potato stem (*Solanum tuberosum* L.). *Bacillus thuringiensis* subsp. *israelensis* B23 and *Escherichia coli* PLG107 served as positive and negative controls, respectively. a: Panel A, Z3-3 alone on plant slices; Panel B, Z3-3 + QL-9a; Panel C, Z3-3 + B23; Panel D, Z3-3 + PLG107. b: Maceration tissue in each treatment. Experimental data were analyzed by one-way analysis of variance (ANOVA), and means were compared by Bonferroni’s multiple comparison test in GraphPad Prism (Version 6.0). Experiments were arranged as a completely randomized design, and *p*-values < 0.05 were considered statistically significant. Figure S5. The mass spectra of the degradation products with the authentic standard compounds of the National Institute of Standards and Technology (NIST, USA) library database. A: *N*-(3-oxohexanoyl)-*L*-homoserine lactone; B: Homoserine lactone; C: Propanoic acid; D: 3-Methyl-butanamide. Figure S6. Antagonism test between *Pectobacterium carotovorum* Z3-3 and *Pseudomonas multiresinivorans* QL-9a. A: Bacterial suspension of strain QL-9a; B: Sterile water. The results showed that no inhibition zone occurred when strain QL-9a and pathogen Z3-3 grew together. Figure S7. Acylase activity test. A: Intracellular enzymes; B: Extracellular enzymes; C: Blank control, distilled water. Table S1. Biochemical characteristics of *Pseudomonas multiresinivorans* QL-9a.
