# Supplementary material for: The Isolate Pseudomonas multiresinivorans QL-9a Quenches the Quorum Sensing Signal and Suppresses Plant Soft Rot Disease
Source: Plants (Basel). 2023 Aug 24;12(17):3037. doi: 10.3390/plants12173037 (PMC10490365; doi:10.3390/plants12173037)
Supplement: Supplementary file 1 [file plants-12-03037-s001.zip › plants-2527621-supplementary.pdf]

The isolate *Pseudomonas multiresinivorans* QL-9a quenches the quorum sensing signal and suppresses plant soft rot disease

Siqi Liu[1,2#], Xixian Zhu[1,2#], Zhenchen Yan[1,2], Hui Liu[1,2], Lian-Hui Zhang[1,2], Wen-Juan Chen[1,2*], Shaohua Chen[1,2*]

[1]National Key Laboratory of Green Pesticide, Guangdong Province Key Laboratory of Microbial Signals and Disease Control, Integrative Microbiology Research Centre, South China Agricultural University, Guangzhou 510642, China;

[2]Guangdong Laboratory for Lingnan Modern Agriculture, College of Plant Protection, South China Agricultural University, Guangzhou 510642, China

[#]These authors contributed equally to this work.

[*]**Correspondence:**

Email: wenjuanchen@scau.edu.cn (W-J.C.); shchen@scau.edu.cn (S.C.).

Tel: +86-20-8528 8229.

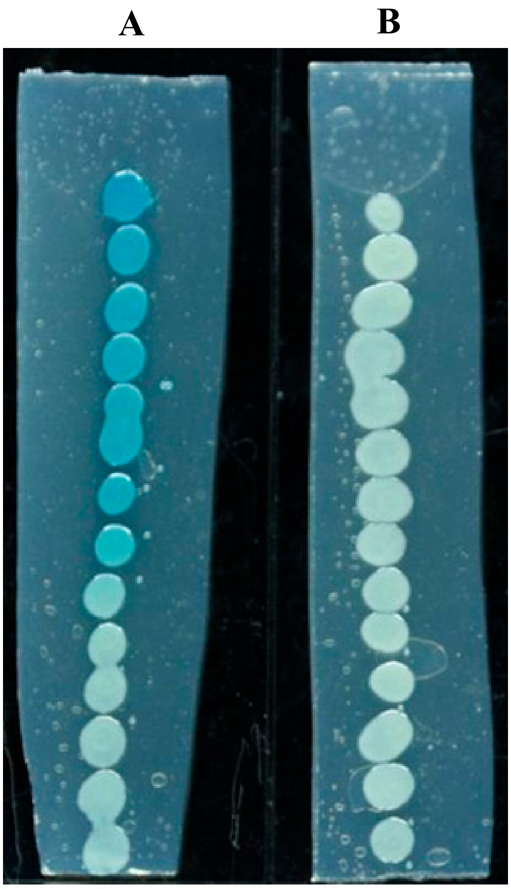

Figure S1. Degradation of *N*-(3-oxohexanoyl)-L-homoserine lactone (OHHL) by strain QL-9a. A: Negative control: only containing OHHL (10 µmol·L^{-1}). B: Degradation of OHHL (10 µmol·L^{-1}) by strain QL-9a. The diffusion length of OHHL and the blue colonies increase with the increase of OHHL concentration. Each colony corresponds to the biosensor strain *Agrobacterium tumefaciens* NT1.

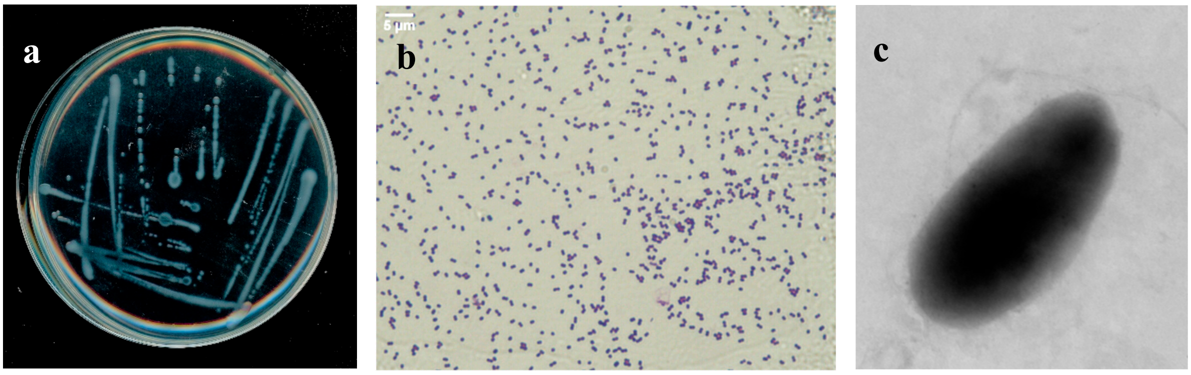

Figure S2. Morphological characteristics of *Pseudomonas multiresinivorans* strain QL-9a. (a) Colonial morphology of strain QL-9a; (b) Cell morphology observed under an optical microscope; (c) Cell morphology observed under the scanning electron microscope (3000×).

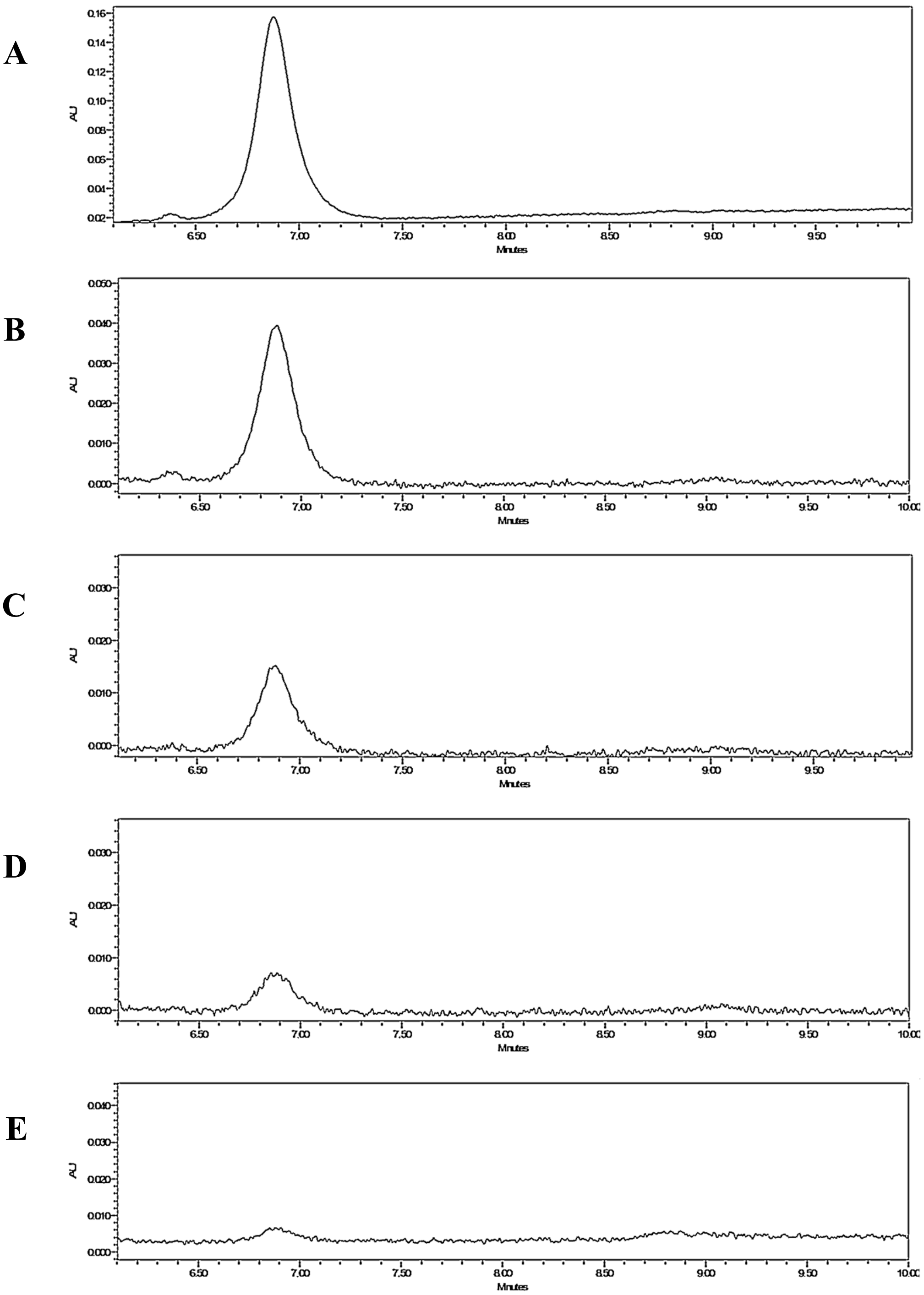

Figure S3. The remaining amount of *N*-(3-oxohexanoyl)-L-homoserine lactone (OHHL) at different time intervals was determined by high-performance liquid chromatography (HPLC): (A) Mineral salt medium (MSM) with OHHL alone as a control. OHHL degradation by the QL-9a strain at 12 h (B), 24 h (C), 36 h (D), and 48 h (E), respectively.

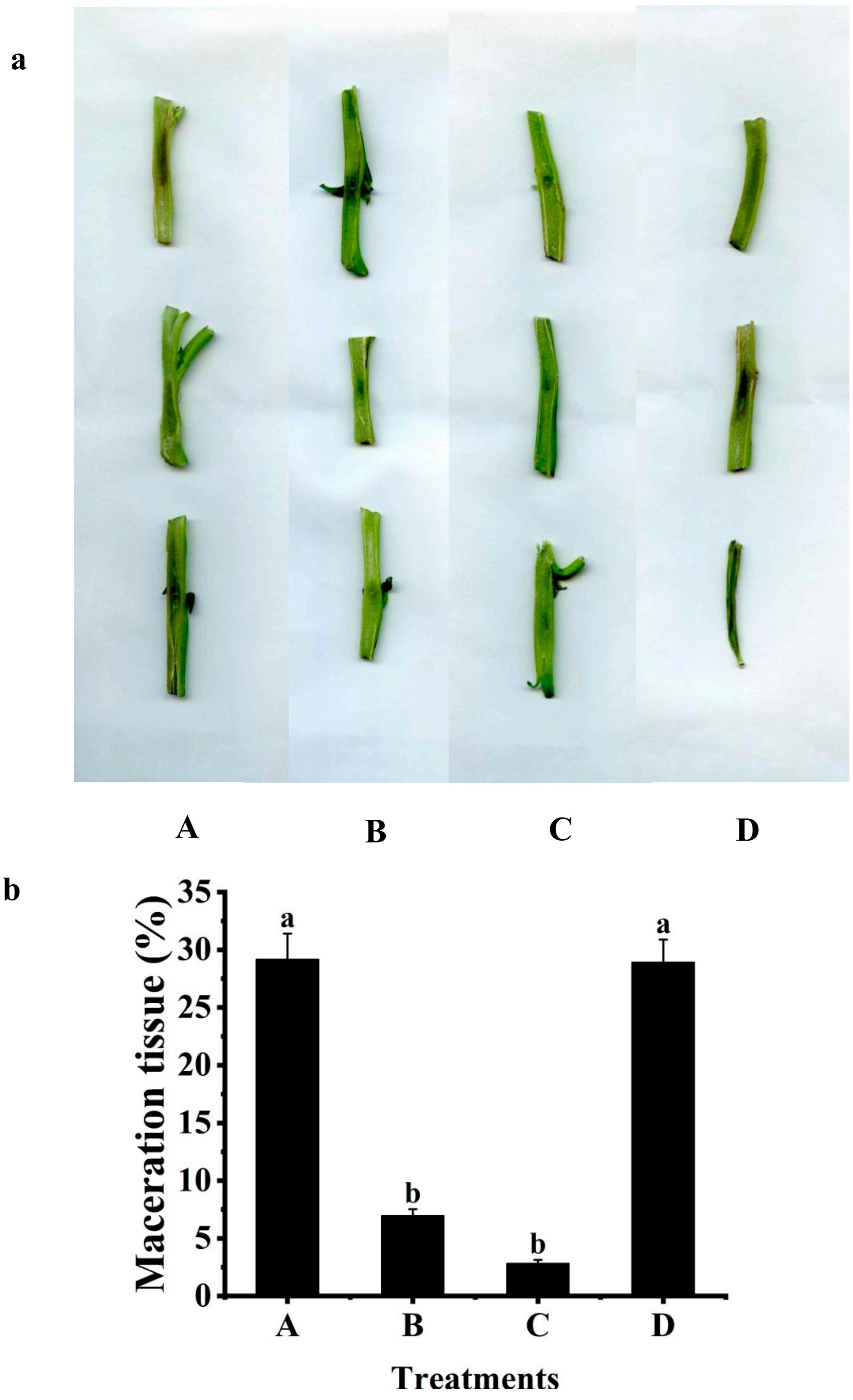

Figure S4. Maceration attenuating test of *Pseudomonas multiresinivorans* QL-9a against soft rot disease pathogen *Pectobacterium carotovorum* Z3-3 on potato stem (*Solanum tuberosum* L.). *Bacillus thuringiensis* subsp. *israelensis* B23 and *Escherichia*

coli PLG107 served as positive and negative controls, respectively. a: Panel A, Z3-3 alone on plant slices; Panel B, Z3-3 + QL-9a; Panel C, Z3-3 + B23; Panel D, Z3-3 + PLG107. b: Maceration tissue in each treatment. Experimental data were analyzed by one-way analysis of variance (ANOVA), and means were compared by Bonferroni's multiple comparison test in GraphPad Prism (Version 6.0). Experiments were arranged as a completely randomized design, and P-values < 0.05 were considered statistically significant.

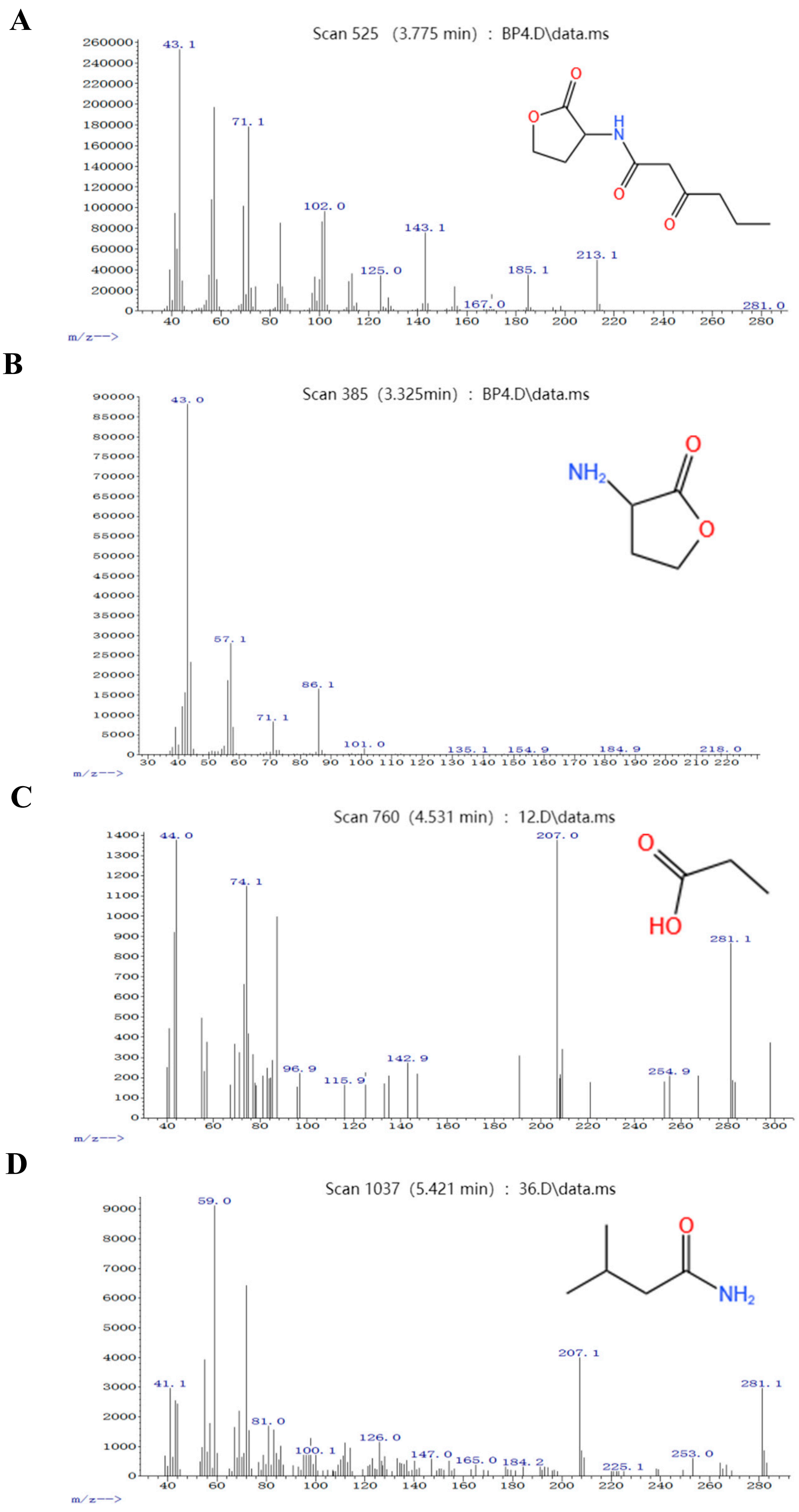

Figure S5. The mass spectra of the degradation products with the authentic standard compounds of the National Institute of Standards and Technology (NIST, USA) library database. A: *N*-(3-oxohexanoyl)-L-homoserine lactone; B: Homoserine lactone, C:

Propanoic acid; D: 3-Methyl-butanamide.

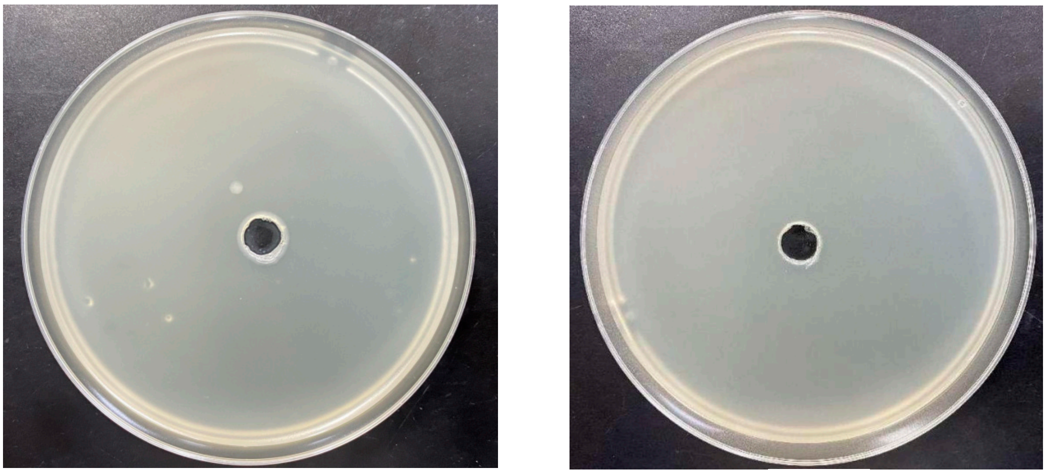

A **B**

Figure S6. Antagonism test between *Pectobacterium carotovorum* Z3-3 and *Pseudomonas multiresinivorans* QL-9a. A: bacterial suspension of strain QL-9a; B: sterile water. The results showed that no inhibition zone occurred when strain QL-9a and pathogen Z3-3 grew together.

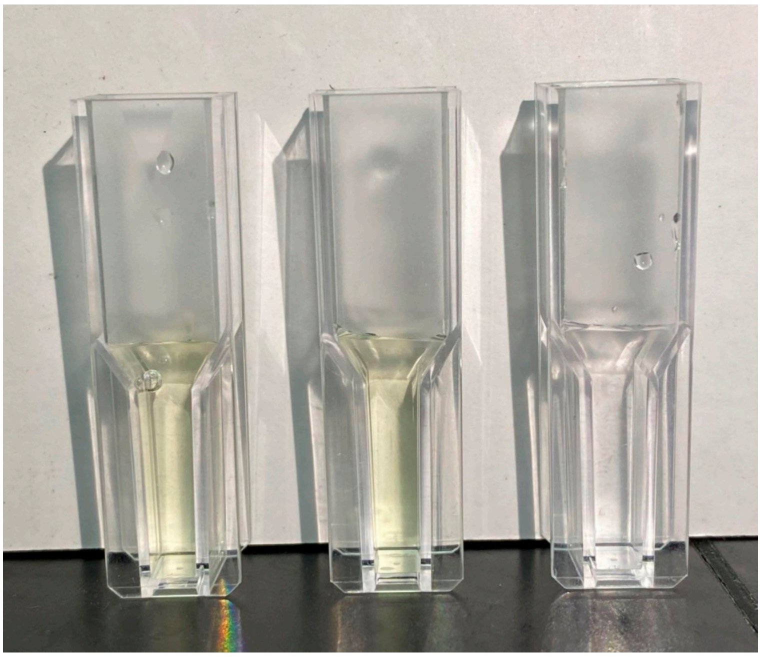

Figure S7. Acylase activity test. A: intracellular enzymes; B: extracellular enzymes; C: blank control, distilled water. Acylase can catalyze the transfer of the acetyl group of acetyl-CoA to butanol and, at the same time, reduce 5,5'-dithiobis-(2-nitrobenzoic acid) (DTNB) to generate 2-nitro-5-thiobenzoic acid (TNB). The TNB compound appears as yellow, with an absorption peak at 412 nm.

Table S1. Biochemical characteristics of *Pseudomonas multiresinivoran* QL-9a

Biochemical characteristics	Results
Oxidase test	+
D-glucose fermentation test	+
Nitrate reduction test	+
Citrate utilization	+
Indole test	—
D-fructose fermentation test	—
V-P assay	—
Methyl red test	—
Hydrogen sulfide test	—

Note: —: negative reaction; + positive reaction.
